# Pd catalyst supported on CeO_2_ nanotubes with enhanced structural stability toward oxidative carbonylation of phenol

**DOI:** 10.1039/c8ra10090j

**Published:** 2019-04-11

**Authors:** Zengjie Fu, Zhimiao Wang, Hongjuan Wang, Fang Li, Wei Xue, Yanji Wang

**Affiliations:** Hebei Provincial Key Laboratory of Green Chemical Technology & High Efficient Energy Saving, Tianjin Key Laboratory of Chemical Process Safety, Hebei University of Technology Tianjin 300130 China lifang@hebut.edu.cn weixue@hebut.edu.cn

## Abstract

Ordered CeO_2_ nanotubes (CeO_2_-T) were prepared *via* a hydrothermal synthesis process using the triblock copolymer polyethylene oxide-polypropylene oxide-polyethylene oxide (P123) as a morphology control agent. CeO_2_-T characterization demonstrated the formation of single crystal structures having lengths between 1–3 μm and diameters < 100 nm. A supported Pd catalyst (Pd/CeO_2_-T) was also prepared through hydrothermal means. H_2_-temperature reduction profile and Raman spectroscopy analyses showed that the oxygen vacancies on the CeO_2_ surface increased and the reduction temperature of the surface oxygen decreased after Pd loading onto CeO_2_-T. Pd/CeO_2_-T was employed as a catalyst toward the oxidative carbonylation of phenol and the reaction conditions were optimized. Phenol conversion was 53.2% with 96.7% selectivity to diphenyl carbonate under optimal conditions. The integrity of the tubular CeO_2_ structure was maintained after the catalyst was recycled, however, both activity and selectivity significantly decreased, which was mainly attributed to the Pd active component significantly leaching during the reaction.

## Abstract 2

Schematic representation of formation of Pd/CeO_2_-T.

## Graphical abstract

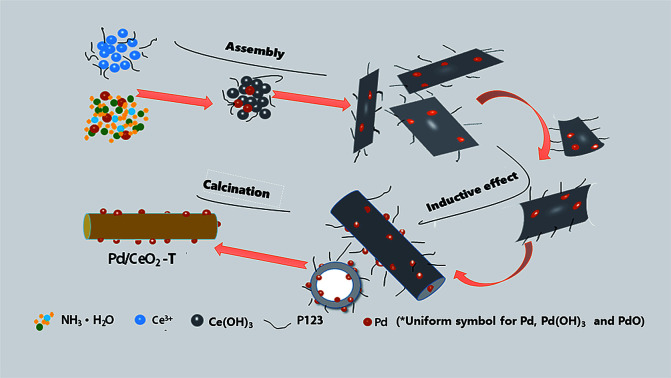

## 1 Introduction

Diphenyl carbonate (DPC) is a significant green organic carbonate that is widely used to synthesize many important organic compounds and polymer materials. Typically, DPC is an important intermediate when producing high quality polycarbonates, which are one of the major thermoplastics engineered to possess excellent mechanical, electrical, optical and heat resistance performance properties.^1,2^ Therefore, the synthesis of DPC has attracted considerable attention. The industrial method to manufacture DPC is based on phosgene, however, because of issues related to the toxicity and corrosive nature of the materials concerned, this manufacturing process will eventually become obsolete. As a more sustainable and green approach, phosgene-free processes have been explored and developed.^2^ Among the phosgene-free routes, oxidative carbonylation of phenol is the most promising candidate, demonstrating several advantages such as readily available starting materials, high atom utilization, and water as the sole by-product.^3^

Hitherto, the main focus of research involving oxidative carbonylation of phenol lies in developing heterogeneous catalysts to enhance repeatability and to facilitate the separation of catalysts from products.^4^ Generally, it is commonly accepted that palladium is the most active species toward the oxidative carbonylation reaction, therefore, a number of heterogeneous palladium catalysts anchored on supports including activated carbon, polystyrene, silicon dioxide, layered double hydroxides, organic–inorganic hybrid materials and mixed metal oxides have been developed.^5^ Zhang *et al.*^6^ reported that the Pd/PbO–MnFe_2_O_4_ catalyst, prepared *via* a co-precipitation method, demonstrated high catalytic activity with a turnover frequency reaching 70.56 mol_DPC_ (mol_Pd_ h)^−1^ toward the oxidative carbonylation of phenol. Xue *et al.*^7^ exploited a water-in-oil microemulsion nanoreactor approach using an embedded Pd–Cu–O/SiO_2_ catalyst giving a DPC yield of 35.4% with reduced Pd leaching and improved stability.

Nonetheless, heterogeneous catalysts are still considered to exhibit less catalytic activity compared with homogeneous catalysts, hence further development of highly efficient heterogeneous catalysts is crucial. The rare earth metal oxide CeO_2_ possesses high oxygen storage capacity and release and strong redox (Ce^3+^/Ce^4+^) performance, and thus is widely used in various oxidation reactions.^8,9^ Interestingly, the reaction performance of CeO_2_ nanostructures is influenced by morphology.^10^ Particularly, Zhou *et al.*^11^ observed that CeO_2_ nanotubes exhibit a larger surface-to-volume ratio than conventional particles because the nanotubes offer two accessible surfaces—internal and external, thus showing enhanced reduction and oxygen storage capacity. Considering Ce as a good redox co-catalyst toward the Pd-catalyzed oxidative carbonylation of phenol, CeO_2_ nanotubes have been studied as a support to promote this reaction. Yuan *et al.*^12^ previously prepared Pd-embedded CeO_2_ polycrystalline nanotubes (CeO_2_-NT) comprising CeO_2_ grains, for the oxidative carbonylation of phenol demonstrating reasonable phenol conversion (67.7%) and DPC selectivity (93.3%). Unfortunately, the structural integrity of the majority of the CeO_2_ nanotubes collapsed into small particles after being subjected to reaction conditions resulting in a substantial reduction in observed catalytic performance. Therefore, to prevent catalyst deactivation, structurally stable CeO_2_ nanotubes is a prerequisite.

In view of the aforementioned issues, this research focused on synthesizing heterogeneous catalysts with an enhanced degree of stability compared with previously reported catalysts. Herein, Pd-supported monocrystalline CeO_2_ nanotube (CeO_2_-T) catalysts possessing well-ordered structures were prepared and their catalyst performance toward the oxidative carbonylation of phenol was investigated.

## 2 Experimental

### 2.1 Preparation of Pd/CeO_2_-T

For CeO_2_-T support preparation, typically, 17.4 g of P123 was dissolved in a mixture of 60 mL absolute ethanol and 60 mL deionized water with ultrasonic treatment and 5.58 g of CeCl_3_·7H_2_O was added to the solution afterwards. With vigorous stirring, a certain concentration of NH_3_·H_2_O solution was added dropwise into the mixture until pH was 10 and then continually stirring for more than 30 min. The resulting suspension was rapidly transferred to the stainless steel autoclave and hydrothermally treated at 160 °C for 72 h. After cooling to room temperature, the solids were separated by centrifugation and washed with water and ethanol until the filtrate was neutral. Finally, the achieved solid was dried at 60 °C and calcined at 500 °C for 4 h with a heating rate of 5 °C min^−1^. The light yellow powder, CeO_2_ nanotubes, was obtained. The sample was denoted as CeO_2_-T.

The preparation of Pd/CeO_2_-T catalyst was similar to the preparation of CeO_2_-T except that a certain amount of aqueous PdCl_2_ solution was added to the suspension before it was transferred to autoclave. The resulting sample was denoted as Pd/CeO_2_-T.

### 2.2 Characterization

Scanning electron microscope (SEM), along with energy dispersive spectrometer (EDS) was performed using Nova Nano SEM 450. Transmission electron microscope (TEM) and selected area electron diffraction (SAED) were observed with PHILIPS TECNOL 20 at an acceleration voltage of 200 kV. Energy dispersive analysis (EDS) of X-ray patterns were collected using FEI Talos F200S transmission electron microscope (TEM). X-ray diffraction (XRD) patterns were recorded on Bruker D8 FOCUS X-ray diffractometer with Cu Kα (40 kV) radiation and a secondary beam graphite monochromator (SS/DS = 1°, RS 0.15 mm, counter SC) at the scanning 2*θ* range of 10–90°. The specific surface areas of the samples were calculated by BET equation using N_2_ adsorption–desorption technique with a Micromeritics ASAP 2020M + C porosity analyzer. Raman spectra were measured by a Renishaw inVia Reflex microspectrometer. Thermo Scientific Escalab 250Xi photoelectron spectrometer (14.6 kV, 200 W) with Al Kα (1486.6 eV) were used for X-ray photoelectron spectroscopy and the number of scanning times was 20 and the correction was performed with C 1s (284.8 eV). The Pd content in the catalyst was determined by a Thermo Scientific iCAP 7400 ICP-OES. Temperature-programmed reduction by hydrogen (H_2_-TPR) was carried out on a Micromeritics AutoChem II-2920 apparatus. All samples (0.1 g) were pretreated in the flow of Ar (50 mL min^−1^) at room temperature for 5 min. Then the flowing gas was switched to 10% H_2_/Ar mixture (50 mL min^−1^) and the sample was heated to 1000 °C at a ramping rate of 10 °C min^−1^. The H_2_ consumption was monitored by a thermal conductivity detector (TCD).

### 2.3 Catalyst activity test

The catalyst activity was evaluated in 50 mL stainless steel autoclave lined with Teflon. The Pd/CeO_2_-T catalyst, cocatalyst (Cu(OAc)_2_, tetrabutylammonium bromide and hydroquinone), desiccant (4A molecular sieve), solvent (CH_2_Cl_2_) and phenol were added to the vessel, sealed and filled with O_2_ and CO. After that, the vessel was heated to the predetermined temperature for reaction. After the reaction was completed, the mixture was cooled to room temperature and the catalyst and 4A molecular sieves were removed by filtration.

Quantitative analysis of the product was performed on a K2600 liquid chromatograph (Knauer, Germany) with a Venusil XBP C18 column (5 μL, 4.6 × 150 mm), CH_3_OH/H_2_O (65/35, v/v) was used as the mobile phase and the detection wavelength was 254 nm. Flow rate 0.6 was mL min^−1^, injection volume was 20 μL and column temperature was kept at 30 °C. External standard method was used to quantify diphenyl carbonate and phenol in the reaction solution.

## 3 Results and discussion

### 3.1 Catalyst characterization

**Fig. 1:**
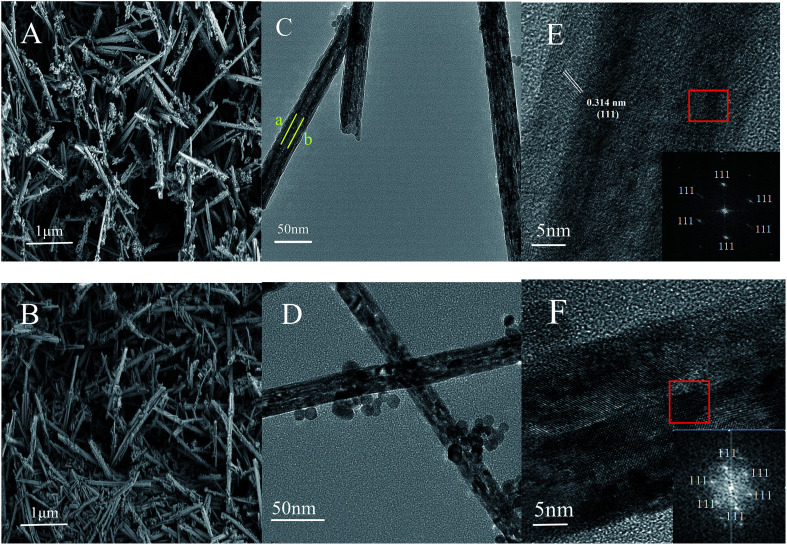
SEM images of: CeO_2_-T (A) and Pd/CeO_2_-T (B). TEM images of: CeO_2_-T (C) and Pd/CeO_2_-T (D), high resolution TEM images and SAED patterns of CeO_2_-T (E) and Pd/CeO_2_-T (F).

CeO_2_-T and Pd/CeO_2_-T morphologies were characterized by scanning electron microscopy (SEM), transmission electron microscopy (TEM) and selected area electron diffraction (SAED). The results are shown in Fig. 1. The CeO_2_-T samples comprise distinct tubes having a length of 1–3 μm. The tubes are 30–60 nm in outer diameter and ∼15 nm in inner diameter, which can be easily recognized in Fig. 1(C). The darker regions in samples marked by lines a and b reveal the internal wall of CeO_2_ nanotubes. Some of the dark regions in TEM image of Pd/CeO_2_-T may be caused by the overlapping of CeO_2_ particles and CeO_2_-T, illustrating the inhomogeneity of the material. Additionally, no distinct changes are observed between the samples before and after Pd loading. However, what is less obvious are the differences observed in the tubular channels that appear in the Pd-loaded catalyst (Fig. 1(D)), when compared with the non-Pd-loaded catalyst (Fig. 1(C)), indicating that the internal structure of the tubes may be influenced as a result of Pd addition. The particles around nanotubes in Fig. 1(C) could be CeO_2_ particles failed to participate in the assembly formation of nanotubes. With the addition of Pd, more particles were formed, shown in Fig. 1(D). This indicates that Pd affects the crystallization process of CeO_2_. SAED patterns of CeO_2_-T and Pd/CeO_2_-T (Fig. 1(E) and (F)), which show the highly active CeO_2_ (111) crystal plane to be exposed, demonstrate that the tubes comprise single crystal structures. Furthermore, Pd particles associated with Pd/CeO_2_-T are not observed from the TEM micrographs, likely as a result of the strong interaction between Pd and CeO_2_. This hinders Pd growth and results in smaller Pd nanoparticles that are highly dispersed within the CeO_2_ lattice.^13^

**Fig. 2:**
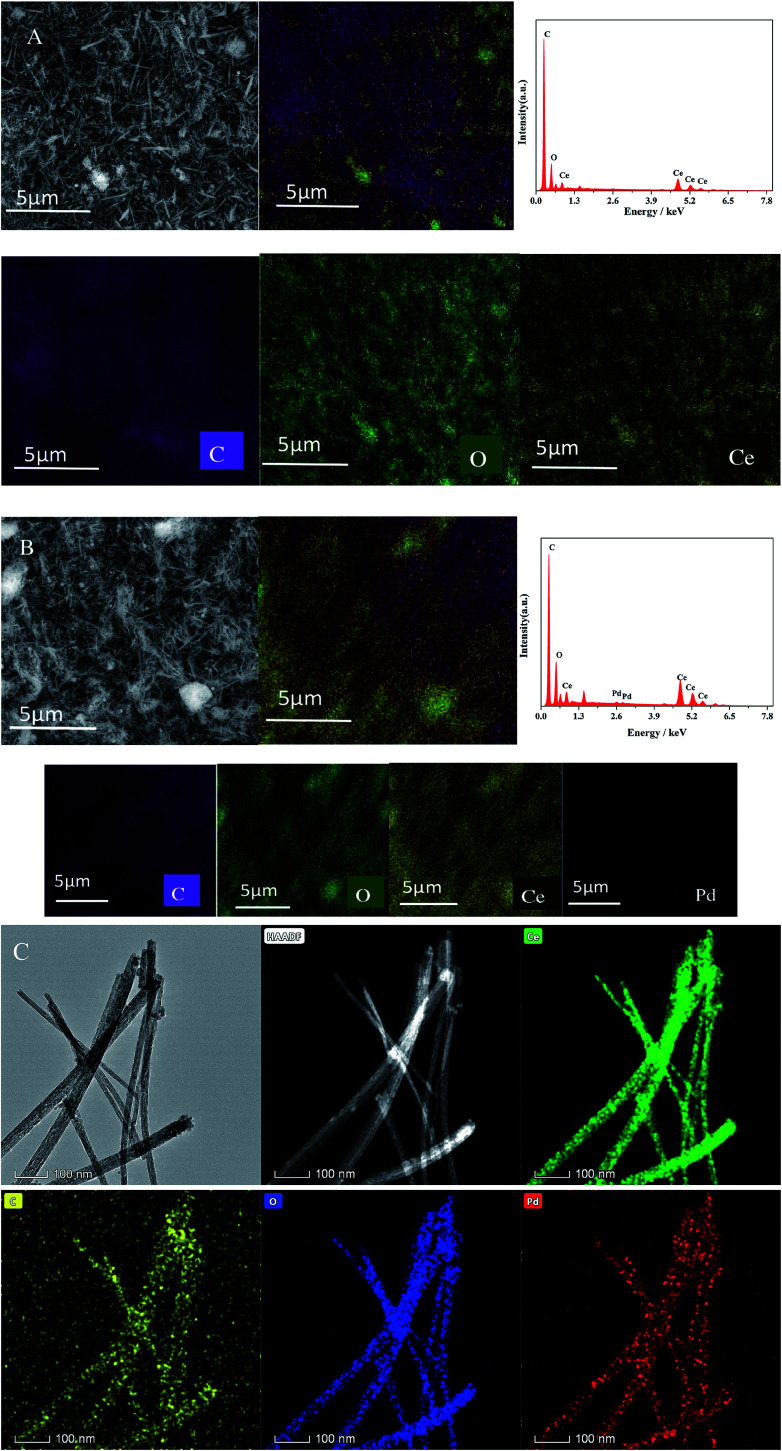
SEM-EDS mapping images of: CeO_2_-T (A) and Pd/CeO_2_-T (B); TEM-EDAX mapping of Pd/CeO_2_-T (C).

SEM-EDS mapping results for CeO_2_-T and Pd/CeO_2_-T are displayed in Fig. 2 and Table 1. CeO_2_-T comprises not only O and Ce, but also high content of C. For Pd/CeO_2_-T catalyst, the same is true. That is because the samples were obtained by calcination in static air. And it was hard to remove completely for P123 under these conditions. Therefore the residual carbon certainly contributed to the C signal. Moreover, part of the C response displayed in SEM-EDS results may come from the conductive glue used for sample preparation. The presence of Pd, in addition to C, O and Ce in Pd/CeO_2_-T demonstrates the successful incorporation of Pd into CeO_2_-T.

**Table 1:** The atom contents and textural properties of CeO_2_-T and Pd/CeO_2_-T

Sample	Content^a^/wt%				*A*_BET_/m^2^ g^−1^	Pore volume/cm^3^ g^−1^	Pore size/nm	Lattice parameter/nm
	C	O	Ce	Pd				
CeO_2_-T	62.60	24.35	13.05	—	56.2	0.18	13.7	0.5412(3)
Pd/CeO_2_-T	59.57	23.08	17.06	0.30	35.5	0.09	22.9	0.5407(4)

a Atomic ratio obtained by SEM-EDS analysis.

**Fig. 3:**
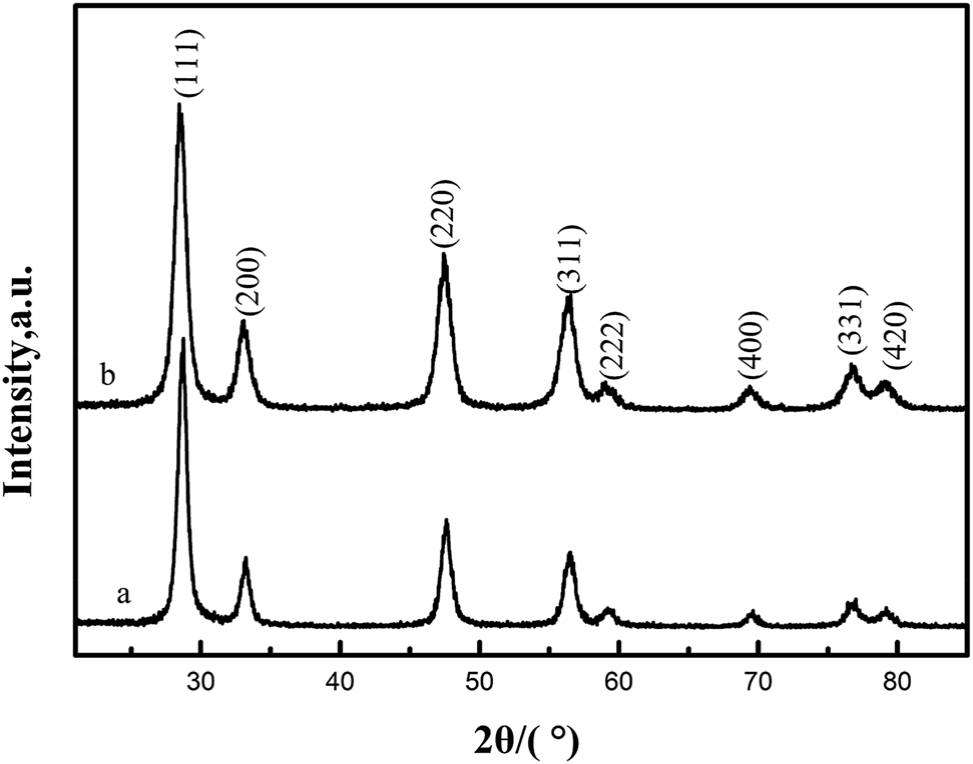
XRD patterns of CeO_2_-T (a) and Pd/CeO_2_-T (b).

X-ray diffraction (XRD) was used to determine phase identification and the crystalline structure. Fig. 3(a) and (b) show the XRD patterns of CeO_2_-T and Pd/CeO_2_-T, respectively. The diffraction patterns show no obvious differences with respect to peak position and strength. The peaks at 2*θ* = 28.54°, 33.09°, 47.57°, 56.48°, 59.03°, 69.41°, 76.75° and 79.11° correspond to the (111), (200), (220), (311), (222), (400), (331) and (420) crystal planes, all of which are well indexed with the cubic fluorite structure of CeO_2_ (JCPDS43-1002). Similar to the TEM micrographs, the characteristic peaks of Pd, or Pd compounds, are not observed either. Two possible reasons accounting for this phenomenon are: Pd exists in an amorphous state; or the Pd particle loading is relatively low and highly dispersed in the support,^14,15^ and thus the peaks cannot be reflected in the diffraction pattern since strong peaks cannot be formed.

The lattice parameters of CeO_2_-T and Pd/CeO_2_-T were calculated from the CeO_2_ (111) plane from the XRD. The lattice parameter of Pd/CeO_2_-T (0.5407 nm) is smaller than that of CeO_2_-T (0.5412 nm). Since the Pd^2+^/Pd^4+^ radii (0.84 Å/0.62 Å) are smaller than that of Ce^4+^ (0.99 Å), it is suggested that Pd in the CeO_2_ lattice replaces Ce^4+^, in part, and induces changes to the internal structure of the catalyst, thereby resulting in a decrease of the CeO_2_ lattice parameter.^16^ A similar phenomenon on the Pd/Ce_0.5_Sn_0.5_O_2_ catalyst was observed by Vasilchenko *et al.*,^17^ when the Ce_0.5_Sn_0.5_O_2_ catalyst was loaded with Pd.

Textural properties of CeO_2_-T and Pd/CeO_2_-T were determined by N_2_ adsorption–desorption measurements, and the results are summarized in Table 1. On the one hand, the structural parameters of CeO_2_ change after Pd loading on CeO_2_-T because Pd influences the crystal growth of CeO_2_. On the other hand, ingress of the Pd species into the pore channels of CeO_2_-T plug the small pores while leaving the relatively larger pores accessible, which increases the pore size of CeO_2_ to 22.9 nm. Previous work has also demonstrated that Pd species in the mesoporous channels of CeO_2_ decreased the specific surface area and pore volume.^18^

**Fig. 4:**
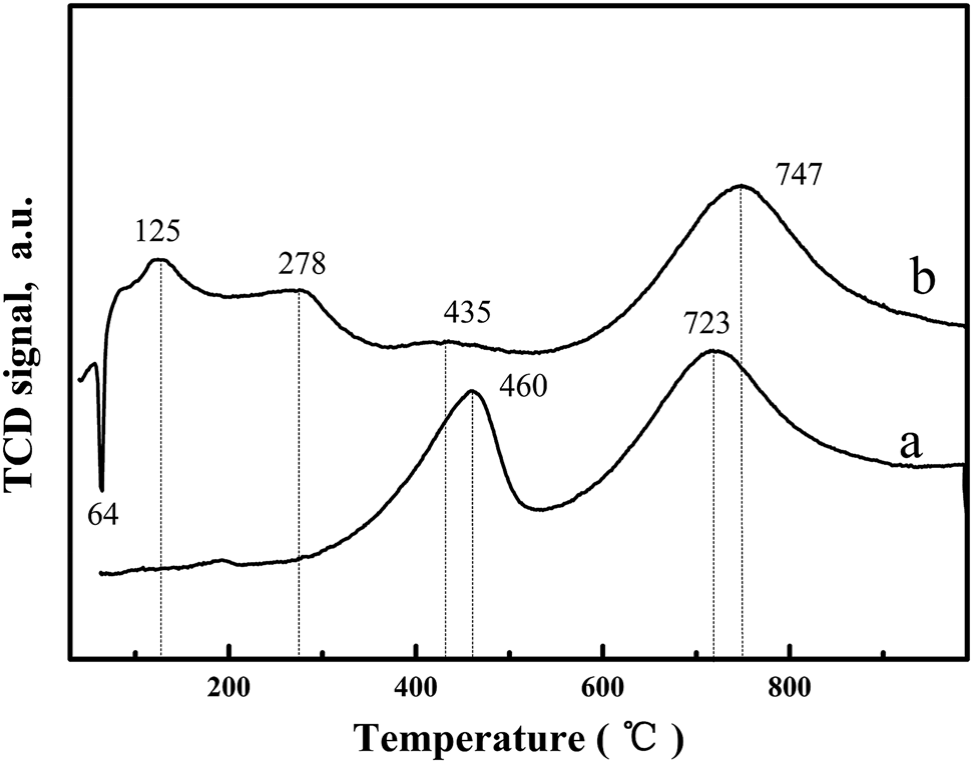
H_2_-TPR profiles of CeO_2_-T (a) and Pd/CeO_2_-T (b).

H_2_-temperature profile reduction (TPR) was undertaken to study the oxygen species reduction capacity and the results are shown in Fig. 4 and Table 2. For CeO_2_-T, the H_2_-TPR profile exhibits two peaks at 460 °C and 723 °C (Fig. 4(a)), corresponding to surface oxygen and bulk oxygen reduction of CeO_2_, respectively.^19^ However, significant changes were observed in the H_2_-TPR curve of Pd/CeO_2_-T (Fig. 4(b)). Regarding the bulk oxygen reduction temperature at 747 °C, the peak position and intensity are similar, however, the reduction temperature of surface oxygen exhibited a weak peak at a decreased temperature of 435 °C (H_2_ consumption is 35.96 μmol g^−1^), and was even accompanied by two low-temperature reduction peaks at 125 °C and 278 °C (The total H_2_ consumption is 389.68 μmol g^−1^). From Table 2, the total hydrogen consumption associated with the three reduction peaks at 125 °C, 278 °C and 435 °C is 425.64 μmol g^−1^, which is similar to the total hydrogen consumption of pure PdO when the reduction temperature is 50 °C (H_2_ consumption is 188 μmol g^−1^)^20^ and surface oxygen on CeO_2_-T reduced at 460 °C (H_2_ consumption is 238.18 μmol g^−1^), indicating that the appearance of the low-temperature reduction peaks is the result of the combined effect of PdO and CeO_2_-T surface oxygen reduction.^21^ Furthermore, there is an inverted peak centered at 64 °C, which demonstrates the high degree of Pd dispersion on CeO_2_-T. Highly dispersed PdO is reduced by H_2_ to form metallic Pd. Thereafter, the Pd crystallites adsorb H_2_, resulting in the formation of palladium hydride (PdH_*x*_), which decomposes as a function of increased temperature releasing H_2_, giving rise to the negative peak.^22^

**Table 2:** H_2_-TPR results of CeO_2_-T and Pd/CeO_2_-T

Sample	Peak temperature (°C)			H_2_ uptake (μmol g^−1^)		
CeO_2_-T	—	460	723	—	238.18	591.71
Pd/CeO_2_-T	125/278	435	747	389.68	35.96	592.42
PdO^20^	50			188		

**Fig. 5:**
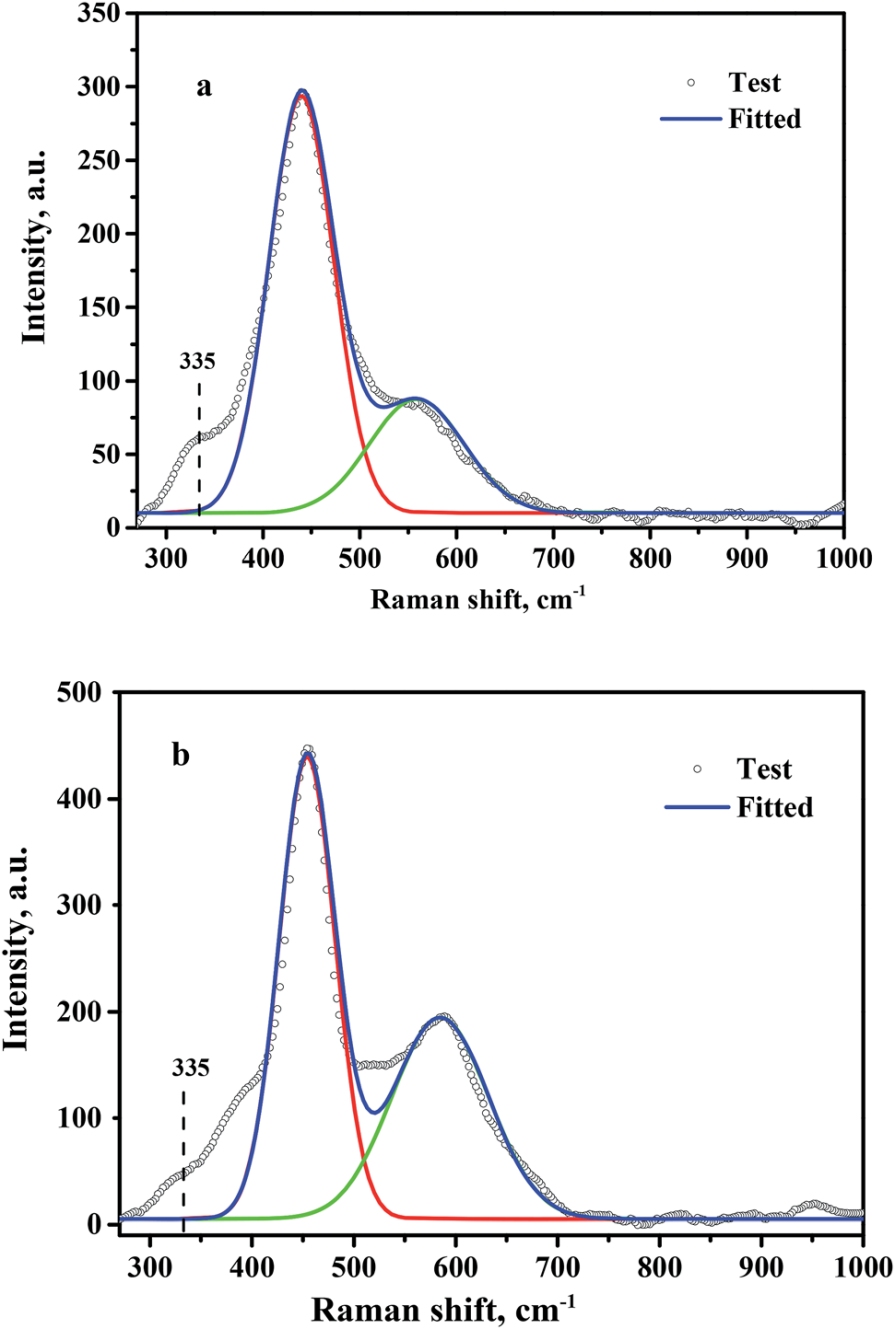
Raman spectra of CeO_2_-T (a) and Pd/CeO_2_-T (b).

Oxygen vacancies present on the CeO_2_ surface were investigated by Raman spectroscopy, and the fitted results are shown in Fig. 5. A strong Raman peak located at 445 cm^−1^ and a relatively weak peak at 572 cm^−1^ were observed for CeO_2_-T (Fig. 5(a)) and Pd/CeO_2_-T (Fig. 5(b)), which correspond to the F_2g_ mode of the ceria fluorite structure and localized vibrations induced by the presence of oxygen vacancies, respectively.^23^ Besides, a shoulder peak at 335 cm^−1^ appears on the side of the fluorite band, but its belonging is unable to determine. Similar cases were reported that a shoulder peak at about 400 cm^−1^ was observed for ceria nanoparticles,^24,25^ and the assignment is also not clear. The density of the oxygen vacancies associated with CeO_2_ in CeO_2_-T and Pd/CeO_2_-T can be expressed by the ratio of *S* (*S* = *S*_1_/*S*_2_),^26^ in which *S*_1_ and *S*_2_ correspond to the peak areas associated with the oxygen vacancies and the F_2g_ vibration. The Raman spectra show that S(Pd/CeO_2_-T) (0.75) is greater than S(CeO_2_-T) (0.40), which suggests that the Pd addition enhances the number of oxygen vacancies in CeO_2_. For Pd/CeO_2_-T, a portion of Pd is incorporated into the CeO_2_ lattice, resulting in a further enhancement to the number of oxygen vacancies on the surface of the support, and molecular oxygen, O_2_, binds to the vacancies easier, which is conducive to the catalytic process.

### 3.2 Oxidative carbonylation of phenol catalyzed by Pd/CeO_2_-T

**Fig. 6:**
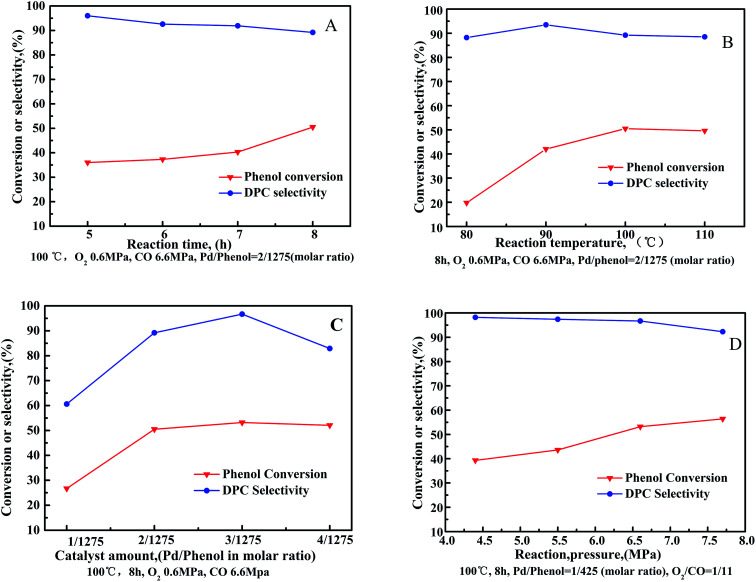
Oxidative carbonylation of phenol over Pd/CeO_2_-T as a function of reaction conditions: (A) reaction time, (B) reaction temperature, (C) catalyst amount, and (D) reaction pressure. Co-catalysts amounts: Pd/Cu(OAc)_2_/TBAB/H_2_BQ = 1/5/40/40 in molar ratio.

The oxidative carbonylation of phenol was investigated as a function of reaction conditions, with the influence of individual parameters such as reaction time, reaction temperature, catalyst amount and reaction pressure shown in Fig. 6.

Phenol conversion increased as a function of reaction time (Fig. 6(A)). Particularly, the rate of phenol conversion was observed to increased sharply between a reaction time of 7–8 h. Conversely, DPC selectivity decreased gradually throughout the entire process. Reactant consumption increases as a function of reaction time, however, water formation also increases, which stimulates the aggregation of Pd nanoparticles resulting in decline of the catalytic activity in target reaction to manufacture DPC.^27,28^ Additionally, decomposition, or self-polymerization, of the resulting DPC occurs as a function of reaction time.^29^

The oxidative carbonylation of phenol increases as a function of reaction temperature (Fig. 6(B)), reflected by increased phenol conversion up to 50.7% at 100 °C. DPC selectivity was observed to increase when increasing the reaction temperature from 80 °C to 90 °C. Further increasing the reaction temperature resulted in a decrease in DPC selectivity. Since oxidative carbonylation of phenol to DPC is an exothermic reaction, the rise in temperature is unfavorable for the reaction to proceed in the desired direction. Furthermore, higher reaction temperatures reduce the solubility of O_2_ and CO, which reduces their contact with the reagents.

The influence of Pd/CeO_2_-T amount on the oxidative carbonylation of phenol is shown in Fig. 6(C). Phenol conversion increased rapidly with increased catalyst loading, reaching a maximum conversion of 53.2% at 1/425(3/1275) Pd/phenol in molar ratio. Further increasing the catalyst loading does not further improve conversion. Catalysts speed up the rate of reaction *via* providing an alternative reaction pathway, nevertheless, an excessive catalyst amount does not offer any benefit because of the mass transfer resistance. DPC selectivity increases first, reaching 96.7% when the system comprises a Pd catalyst/phenol molar ratio of 1/425. Further increase the catalyst loading results in a decrease in DPC selectivity. Lower catalyst concentrations can enhance the active sites to promote catalysis of the main reaction, whereas at high catalyst concentrations, the interactions between the additives and active ingredients are hindered.

The effect of reaction pressure on the oxidative carbonylation of phenol was examined (Fig. 6(D)). The CO partial pressure was fixed at 92 vol% (O_2_/CO = 1/11) to prevent an explosive atmosphere from forming. Phenol conversion increased as a function of increased gas pressure, reaching 56.4% at 7.7 MPa. DPC selectivity decreased slowly from 98.2% at 4.4 MPa to 92.3% at 7.7 MPa. By increasing the gas pressure, the adsorption of CO and O_2_ onto the catalysts is strengthened. The oxidative carbonylation reaction, as a volume-reduced reaction, is easily promoted at higher pressures, however, increased pressures favor undesired side reactions resulting in the transformation of CO to CO_2_, prompting DPC selectivity to decrease.

### 3.3 Catalyst stability

**Fig. 7:**
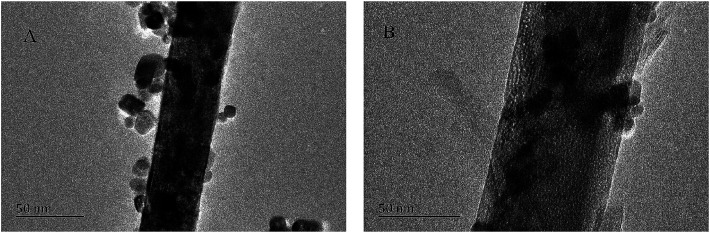
TEM micrographs of Pd/CeO_2_-T (A) and Pd/CeO_2_-TC (B).

**Fig. 8:**
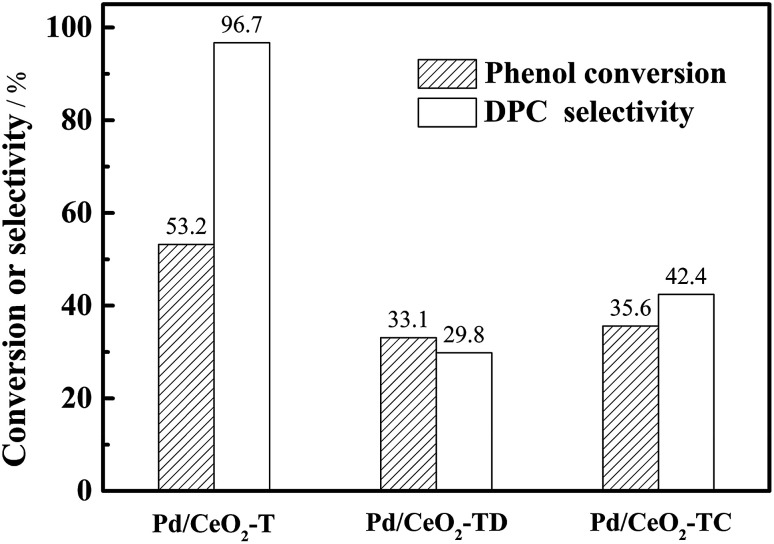
Reusability of Pd/CeO_2_-T catalysts. 100 °C, 8 h, O_2_ 0.6 MPa, CO 6.6 MPa, Pd/phenol = 1/425 (molar ratio), co-catalyst amounts: Pd/Cu(OAc)_2_/TBAB/H_2_BQ = 1/5/40/40 in molar ratio.

**Fig. 9:**
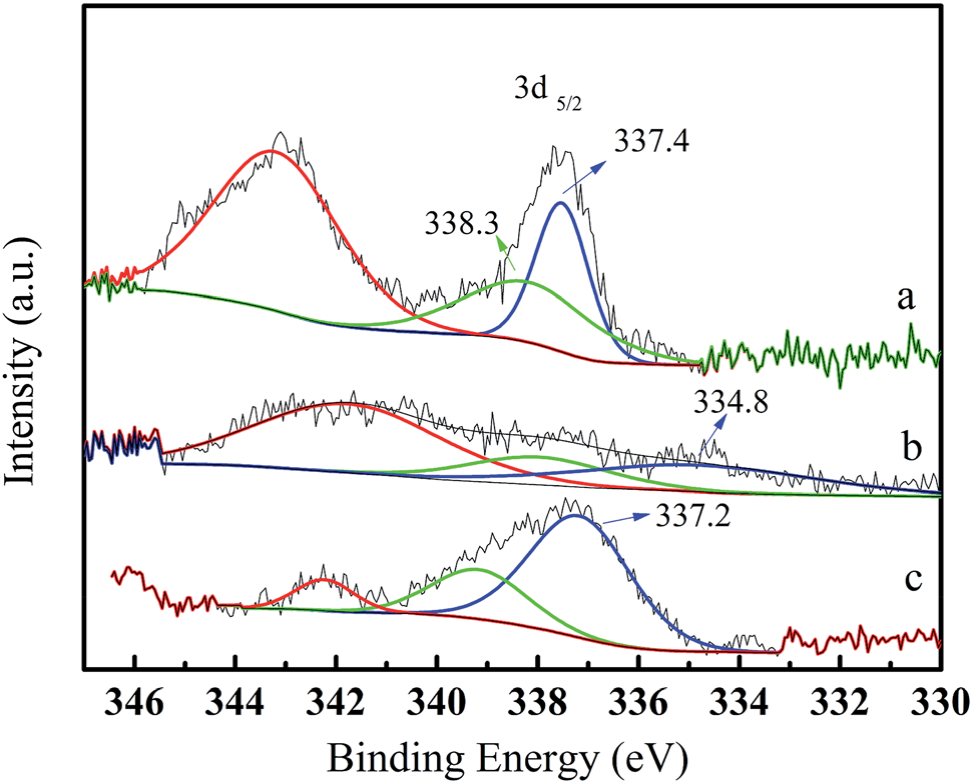
X-ray photoelectron spectroscopy Pd 3d spectra of fresh and recovered Pd/CeO_2_-T catalysts. Pd/CeO_2_-T (a); Pd/CeO_2_-TD (b); Pd/CeO_2_-TC (c).

The reusability of the Pd/CeO_2_-T catalyst was examined. After the reaction, the catalyst was recovered, washed with ethanol and dried at 60 °C, and referred to as Pd/CeO_2_-TD. Subsequently, the dried sample was calcined at 500 °C for 1 h and termed Pd/CeO_2_-TC. TEM micrographs (Fig. 7) and catalytic performance data (Fig. 8) of Pd/CeO_2_-T, before and after the reaction, provide insight into the catalyst stability. The structural integrity of the single crystal nanotubes is maintained in the recovered catalyst. Phenol conversion and DPC selectivity on the recovered catalysts, regardless of the treatment methods, were significantly reduced. X-ray photoelectron spectroscopy (XPS) was performed on the three catalysts to determine the Pd state, and the results are summarized in Fig. 9. The Pd 3d_5/2_ binding energy of Pd/CeO_2_-T is 337.4 eV, ascribed to PdO, whereas the peak located at 338.3 eV is assigned to Pd_*x*_Ce_1−*x*_O_2._^30,31^ The formation of Pd_*x*_Ce_1−*x*_O_2_ results from the electron transfer from Pd to Ce, giving rise to Ce having a surplus electron density (Ce^*δ*−^) and an electron-deficient Pd species (Pd^*δ*+^), that induces a strong interaction between Pd and CeO_2_, which increases the binding energy of PdO.^32^ In the Pd/CeO_2_-TD spectrum, the peaks corresponding to PdO and Pd^*δ*+^ are very weak, while the 3d_5/2_ peak at 334.8 eV, corresponding to Pd^0^ is detected, indicating the reduction of Pd(ii) to Pd(0). This transformation from Pd(ii) to Pd(0) leads to catalyst deactivation.^5^ Additionally, the peaks corresponding to PdO and Pd^*δ*+^ in the Pd/CeO_2_-TC spectrum become stronger, whereas the 3d_5/2_ peaks associated with Pd(0) almost disappear, which is as a result of Pd^0^ re-oxidation during calcination. Notably, the catalytic activity toward the oxidative carbonylation of phenol of the recycled Pd/CeO_2_-TC catalyst is a little higher than the Pd/CeO_2_-TD catalyst even though the majority of Pd^0^ was reactivated into bivalent Pd species. We inferred that valent variation of Pd is not the main factor for catalyst deactivation. The Pd content was determined by inductively coupled plasma (ICP) analysis. The results show that the Pd content in Pd/CeO_2_-T, Pd/CeO_2_-TD and Pd/CeO_2_-TC is 1.87 wt%, 0.13 wt% and 0.23 wt%, respectively. Significant Pd leaching is believed to be the main factor in catalyst deactivation of Pd/CeO_2_-T.

In addition to the observed enhanced reaction rate and selectivity, the nanotube structural integrity of the obtained single crystal Pd-supported CeO_2_ nanotubes is retained after being subjected to the oxidative carbonylation of phenol reaction, which is a distinguishing feature from the polycrystalline Pd–O/CeO_2_-NT catalyst.^12^ However, the reutilization of Pd/CeO_2_-T is not satisfactory, as only a 35.6% phenol conversion was observed with DPC selectivity at 42.4%—lower than the DPC conversion and selectivity observed for Pd–O/CeO_2_-NT (29.3% phenol conversion with 62.3% DPC selectivity).^12^ Pd loss is not a negligible factor in catalyst deactivation from the observed relationship between weak activity and significant Pd leaching in recycled Pd/CeO_2_-T. Therefore, to improve catalyst performance, it is of great interest to maintain the structural integrity of the support and to limit Pd loss. Yin *et al.*^5^ suggested that the solvent has a significant impact on Pd leaching in Pd/La_0.5_Pb_0.5_MnO_3_ during the oxidative carbonylation of phenol. The leached palladium content in the post-reaction filtrate reached 3.51% in dichloromethane and 8.90% in dimethylformamide. In the non-solvent system, Pd leaching was only 0.13%. Hence, a test matrix studying the influence of solvent selection should be considered in detail. Additionally, another point of interest is that although the recovered Pd/CeO_2_-T catalyst has a lower Pd content, phenol conversion is higher than Pd–O/CeO_2_-NT. The catalysts prepared herein demonstrate the importance of the CeO_2_ nanotube structures to maintain the CeO_2_ function as a co-catalyst and to enhance catalytic performance. Therefore, monocrystalline CeO_2_ nanotubes with ordered structures demonstrate great potential as catalysts and warrant further research to design new materials.

## 4 Conclusions

In summary, CeO_2_-T with uniform tubular morphology of 1–3 μm were synthesized *via* a hydrothermal method using P123 as a surfactant to control morphology. The CeO_2_ (111) crystal plane was observed to be exposed in the CeO_2_-T support, suggesting the formation of single crystal structures. The Pd/CeO_2_-T catalyst was prepared based on the CeO_2_-T support. Characterization data showed that active palladium species were incorporated into the CeO_2_ lattice that resulted in a synergistic effect with CeO_2._ Catalytic performance was observed to improve after Pd loading as a result of increased oxygen vacancies on the CeO_2_ surface together with a decrease in the surface oxygen reduction temperature. The oxidation carbonylation of phenol was performed in the presence of Pd/CeO_2_-T, with phenol conversion reaching 53.2% and DPC selectivity at 96.7% when subjected to the following reaction parameters: 100 °C, 8 h, a Pd/phenol molar ratio of 1/425, and a CO pressure of 6.6 MPa. Although significant Pd leaching from Pd/CeO_2_-T was observed that resulted in activity loss, the structural integrity of the tubular monocrystalline Pd/CeO_2_-T was maintained during the reaction, which is a key factor to enhanced catalytic performance.

## Conflicts of interest

There are no conflicts to declare.

## Supplementary Material
